# The effects of male age on sperm DNA damage: an evaluation of 2,178 semen samples

**DOI:** 10.5935/1518-0557.20180047

**Published:** 2018

**Authors:** Claudia G. Petersen, Ana L. Mauri, Laura D. Vagnini, Adriana Renzi, Bruna Petersen, Mariana Mattila, Vanessa Comar, Juliana Ricci, Felipe Dieamant, Joao Batista A. Oliveira, Ricardo L. R. Baruffi, Jose G. Franco Jr.

**Affiliations:** 1 Center for Human Reproduction Prof. Franco Jr., Ribeirão Preto, SP, Brazil; 2 Paulista Center for Diagnosis, Research, and Training, Ribeirão Preto, SP, Brazil

**Keywords:** Male age, DNA damage, sperm, functional parameters

## Abstract

**Objective:**

This study aimed to evaluate the effects of male age on sperm DNA damage.

**Methods:**

This cross-sectional study included semen samples collected from 2,178 men seen at an infertility clinic. For DNA integrity analysis, the proportions of spermatozoa showing DNA fragmentation (TUNEL assay), abnormal chromatin packaging/underprotamination (chromomycin A_3_), abnormal mitochondrial membrane potential (MMP/MitoTracker Green), and apoptosis (annexin V) were recorded. For group comparisons, enrolled subjects were divided into three groups based on their ages: ≤35 years; 36-44 years; and ≥45 years. The associations between age and sperm parameters were assessed using Spearman's rank correlation coefficient.

**Results:**

Although aging did not affect sperm apoptosis (*p*>.05), sperm DNA fragmentation and MMP deteriorated significantly with age (*p*<.05). Chromatin packaging/protamination improved significantly with age (*p*<.05).

**Conclusion:**

Sperm DNA fragmentation worsened with age and was apparently associated with mitochondrial damage. The age-related increase in sperm DNA damage suggests that delaying childbearing, not only in women but also in men, might jeopardize a couple’s reproductive capacity. The increase seen in chromatin packaging might represent a protective feature for DNA. However, additional studies must be performed to confirm the results concerning chromatin packaging/protamination.

## INTRODUCTION

The hormonal and cellular changes introduced by the aging process affect human fertility. Understanding the effect of age on fertility is important, since couples in today’s society frequently postpone parenthood. The female biological clock has been established as having a negative effect on fertility. However, with respect to evaluating men in terms of this concern, the effects of male aging have not been established, although increasing evidence suggests that advanced paternal age affects fertility, independent of maternal age (Andolz *et al*., 1999; Girsh *et al*., 2008; Dain *et al*., 2011; Zhu *et al*., 2011). The general consensus is that increasing paternal age tends to be associated with a decline in semen quality with respect to basic, structural, and functional sperm parameters. Several studies have established that semen volume and motility decrease in 50-year-old men (Neaves *et al*., 1984). However, studies analyzing male age have demonstrated declines in structural and functional parameters and established the age of 40 as a cutoff or turning point (Guérin *et al*., 2005; Evenson & Wixon, 2006; Belloc *et al*., 2008). Additionally, studies have indicated that advanced paternal age increases the risk of miscarriage (Slama *et al*., 2005; Kleinhaus *et al.*, 2006) and the potential of certain diseases occurring in the offspring, such as genetic abnormalities, pediatric cancer, and several neuropsychiatric disorders (Hemminki & Kyyrönen, 1999; Wyrobek *et al*., 2006; O’Roak *et al*., 2012; Crosnoe & Kim, 2013; Paul & Robaire, 2013). Advanced male age has also been correlated with infant mortality (Urhoj *et al*., 2014). One likely explanation for these outcomes is that older men may have more sperm with damaged DNA.

The exact mechanism for age-dependent patterns of sperm decline is still not fully understood. Several factors such as free radical theory and apoptosis, fertilization capacity, DNA mutation, other diseases, and changes in telomeres have been discussed in the literature. It has been scarcely suggested that oxidative stress and reactive oxygen species (ROS) are important contributors for both damage to the DNA of the nucleus and of the mitochondria and decrease in sperm motility. Increased age causes an accumulation of ROS, promoting increased oxidative stress that induces lipid peroxidation and further ROS generation. An excessive amount of ROS and decreased antioxidant capacity in the course of aging may induce apoptosis or oxidative damage to DNA (Aitken, 1989). The damaged paternal DNA, if not repaired, may through fertilization reach the couple’s offspring, causing a variety of diseases (Gunes *et al*., 2016). Older men are believed to produce more sperm with DNA damage, gene mutations, and aneuploidy (Nijs *et al*., 2011; Plastira *et al*., 2007). A systematic review and meta-analysis (Johnson *et al*., 2015) suggested that greater focus on DNA fragmentation and progressive motility in a clinical setting might produce better patient outcomes during fertility treatments of aging couples.

To better understand sperm quality and function, a variety of methods, such as tests to quantify protamination and DNA packaging, DNA fragmentation, chromosome aneuploidy, and molecular karyotyping, have been applied in the evaluation of infertile males (Patassini *et al*., 2013; Ferlin & Foresta, 2014; Tsuribe *et al*., 2016). However, only a few studies have described semen cytochemical parameters such as apoptotic and DNA mitochondrial damage. The objective of this study was to evaluate the correlations between male age and four sperm biomarkers - DNA fragmentation, chromatin packaging, apoptosis, and mitochondrial damage - in a large population.

## MATERIAL AND METHODS

### Study Participants

This prospective study was based on a cohort of 2178 consecutive men from couples undergoing infertility investigation and treatment from January 2007 to December 2015. The exclusion criteria were azoospermia, any known reproductive tract pathology in the last six months, any hormonal therapy in the last six months, chronic medical disorders, congenital genital tract abnormalities or previous treatment that might have affected fertility (cancer treatment). The participants gave written consent to joining the study. The local Institutional Review Board approved the study.

### Sample collection

Semen samples were collected in sterile containers by masturbation after a sexual abstinence period of 2–5 days. A portion of each sêmen sample was used for analysis according to the WHO guidelines (WHO, 2010).The other portion of each semen sample was immediately processed for morphological analysis by motile sperm organelle morphology examination (MSOME). The remainder of the semen samples was immediately processed for sperm DNA fragmentation analysis using the TdT (terminal deoxynucleotidyl transferase)-mediated dUTP nick-end labelling (TUNEL) assay, sperm apoptosis analysis using the annexin V assay, sperm chromatin packing/protamination using chromomycin A3 (CMA_3_) staining and sperm mitochondrial membrane potential (MMP) using MitoTracker Green FM.

### Cytochemical Evaluation

#### Determination of sperm DNA fragmentation/TdT-mediated dUTP TUNEL

DNA fragmentation in spermatozoa was measured using the TUNEL assay, performed using an in situ cell death detection kit and tetramethylrhodamine-red labelled dUTP (Roche), as previously described (Vagnini *et al*., 2007; Oliveira *et al*., 2014). The final evaluation was performed using a fluorescence microscope (Olympus BX 50), and the proportion of TUNEL-positive spermatozoa was determined. At least 200 sperms per slide were evaluated, using the appropriate filter.

#### Determination of sperm chromatin packaging/protamination/chromomycin A_3_ (CMA_3_) staining

Sperm protamine deficiency (underprotamination)/chromatin packaging was measured using CMA_3_ (Sigma-Aldrich), as previously described (Franco *et al*., 2012). The proportion of positive spermatozoa was determined by direct observation in four fields on a fluorescence microscope (Olympus BX 50), and the proportion of spermatozoa with abnormal chromatin packaging was determined. At least 200 sperms per slide were evaluated, using the appropriate filter.

#### Determination of sperm apoptosis/annexin V binding

Sperm apoptosis was measured using annexin V, a calcium-dependent phospholipid-binding protein with a high affinity for phosphatidylserine that is present in the inner leaflet of the sperm membrane, except in apoptotic sperm, where phosphatidylserine is externalized. The sperm suspensions (1 × 10^6^ cells/mL) were incubated in an appropriate binding buffer with 1 µL of annexin V (green), 1 µL of propidium iodide (PI) (red) (Dead Cell Apoptosis Kit with Annexin V Alexa Fluor^®^ 488 & Propidium Iodide, Molecular Probes^TM^, Eugene, OR) and 1 µL of cell-permeable DNA stain Hoechst 33342 (blue) (Molecular Probes) at room temperature for 15 min in the dark. PI is impermeable to live cells. After incubation, the suspension was centrifuged at 800 *g* for 10 min, and the pellet was mounted on poly-l-lysine-coated slides for examination, using a fluorescence microscope (Olympus BX 50). The results demonstrated that subpopulations of sperm could be identified: annexin V(−)/PI(−) - live intact sperm; annexin V(+)/PI(−) - early apoptotic cells; and annexin V(+/−)/PI(+) - necrotic cells. The proportion of early apoptotic cells (defined as the number of positive annexin V/negative PI spermatozoa divided by the total number of spermatozoa ×100) was determined. At least 200 sperms per slide were evaluated, using the appropriate filter.

#### Determination of sperm MMP - MitoTracker Green (MT-G) staining

Sperm MMP, an indicator of sperm mitochondrial functionality, was determined using MG FM (Molecular Probes). The live sperm suspensions were incubated in phosphate-buffered saline (PBS) containing 20 nmol/L MG for 20 min at 37ºC. To stain sperm DNA, the samples were subsequently incubated in cell-permeable DNA stain Hoechst 33342 (Molecular Probes) for 10 min at 37ºC. After incubation, the suspension was centrifuged at 800 *g* (10 min) and the pellet was mounted on a microscope slide. Green fluorescence in the midpiece indicated active mitochondria. Sperm samples were examined using a fluorescence microscope (Olympus BX 50) and the proportions of spermatozoa with altered MMP/mitochondrial damage (i.e., absence of green fluorescence) were determined. At least 200 spermatozoa per slide were evaluated, using the appropriate filter.

### Quality control

To control for intra-observer and inter-observer variability, multiple fractions of semen samples were obtained from randomly selected patients. Each sample was observed at least three times by the same observer (blinded to subject identity). Intra-observer and Inter-observer variations of ≈0.5% to 1% and 0.5% to 7%, respectively, were obtained for each parameter analyzed: TUNEL-positive sperm, CMA_3_-positive sperm, annexin V-positive sperm and MitoTracker Green-positive, sperm semen parameters (according to the WHO guidelines), normality of the spermatozoon (as a whole), and normality of the nuclear structure. The variability observed here was comparable to the variability described in classical sperm quality parameters (Auger *et al*., 2000).

### Sample size

The sample size was calculated by performing a comparison between two proportions. A sample size of 300 subjects in each group yielded a chance of 80% of detecting an increase of 10% with a significance level of 0.05 (two-tailed).

### Statistical analysis

The data were analyzed using software package StatsDirect (Cheshire, UK). Potential confounders - Body Mass Index (BMI), abstinence time, smoking, alcohol, varicocele, and vitamin use - were also assessed. Regression and correlation analyses with continuous variables were performed using Spearman's rank correlation coefficient. For dichotomous variables, the correlations were determined using logistic regression.

The following age ranges were used as cutoff points to divide the subjects into groups: Group 1: ≤35 years; Group 2: 36-44 years; and Group 3: ≥45 years. The Mann-Whitney U test, Student’s *t*-test and chi-squared test were used when indicated.

The level of significance was set at *p*<0.05.

## RESULTS

Table 1 shows the characteristics of the male study population.

**Table 1:** General characteristics of the male study population.

Characteristics	Total
Patients (n)	2178
Age (years) (mean±SD)	37.9±6.4 (min, 22; max, 76)
Father of at least one child (%)	32.8 (715/2178)
Duration of infertility (years) (mean±SD)	4.1±2.2
BMI (mean±SD)	28.5±4.2
Smoking (%)	11.2 (243/2178)
Regular drinking (%)	67.2 (1463/2178)
Vitamin supplement use (%)	16.7 (364/2178)
Varicocele (%)	16.0 (349/2178)
Sperm cytogram Sexual abstinence (days) (mean±SD) -pH (mean±SD) -Volume (mL) (mean±SD) -Concentration (×10^6^/ml) -Motility (%) (mean±SD) Total Progressive (rapid+slow) -Morphology (MSOME): normal sperm forms (%) (mean±SD) -Leucocytes /×10^6^/mL (mean±SD) -Vitality (%) (mean±SD)	3.5±1.5 8.0±0.5 2.7±1.4 74.6±61.6 63.7±15.7 56.9±16.2 0.9±1.5 0.4±1.0 65.0±14.5

### General characteristics of the male population and age (Table 2)

Table 2 shows the correlation between male general characteristics and age. A significant (*p*<0.0001) positive correlation was found between age and two characteristics: being a father with at least one child (OR: 1.08) and duration of infertility (r: 0.26). Other characteristics such as the BMI, smoking, drinking, intake of vitamin supplements, varicocele, and abstinence days, were not correlated with age (*p*>0.05). The proportion of men who fathered at least one child increased with age. A significantly higher number (%) of men aged ≥45 years (Group III) had fathered at least one child, compared to the number of men aged 36−40 years (Group II) and men aged ≤35 years (Group I) - 53.5%, 34.4%, and 23.4%, (*p*<0.05), respectively. Similarly, men aged ≥45 years presented a larger period of infertility when compared to men aged 36-40 years or ≤35 years - 5.6, 4.4, and 3.1 (*p*<0.05), respectively.

**Table 2:** Correlation between male general population characteristics and age.

	Age Groups				Regression Analysis		
Characteristics	Group 1 (≤35 years)	Group 2 (36−44 years)	Group 3 (≥45 years)	*p*	Correlation Coefficient r/OR	95% Cl	*p*
Patients (n)	852	1014	312				
Age (years) (mean±SD)	32.1±2.6	39.3±2.5	49.2±5.0				
Father of at least one child (%)	23.4 (199/852)	34.4 (348/1014)	53.5 (167/312)	<0.05	OR: 1.08^••^	1.06 to 1.09	<0.0001
Duration of infertility (years) (mean±SD)	3.1±1.1	4.4±2.1	5.6±3.3	<0.05	r: 0.26^•^	0.21 to 0.29	<0.0001
BMI (mean±SD)	28.6 ± 4.3	28.5 ± 4.3	28.4±4.2	0.83	r: 0.03^•^	-0.07 to 0.02	0.25
Smoking (%)	11.5 (98/852)	11.0 (112/1014)	10.6 (33/312)	0.22	OR:0.99^••^	0.97 to 1.01	0.45
Regular drinking (%)	68.9 (587/852)	65.7 (666/1014)	67.3 (210/312)	0.33	OR:0.99^••^	0.98 to 1.01	0.95
Vitamin supplement use (%)	16.5 (14/852)	16.0 (162/1014)	19.6 (61/312)	0.39	OR: 1.01^••^	0.99 to 1.03	0.12
Varicocele (%)	14.7 (125/852)	17.6 (178/1014)	14.7 (46/312)	0.19	OR: 1.00^••^	0.98 to 1.01	0.84
Sexual abstinence (days) (mean±SD)	3.4±1.0	3.5±1.8	3.6±1.6	0.58	r:0.03	-0.02 to 0.07	0.23

* Spearman’s correlation;

** logistic regression; r, Spearman’s rank correlation coefficient; OR, odds ratio; CI, Confidence Interval.

### Cytochemical sperm parameters

The overall percentage of DNA fragmentation was 15.4±8.5%; chromatin packaging was 56.1±15%; mitochondrial damage was 25.9%; and the proportion of apoptotic cells was 19.2% (Table 3).

**Table 3:** Cytochemical sperm parameters of 2178 patients divided between the three men’s age groups.

Cytochemical Sperm Parameters	Age Groups				
	Total	Group 1 (≤35 years)	Group 2 (36−44 years)	Group 3 (≥45 years)	*p*
	n=2178	n=852	n=1014	n=312	
DNA fragmentation (%) (mean±SD)	15.4 ±8.5	14.7±8.3^a,b^	15.9±8.7^a^	16.2±8.4^b^	^a^0.002 ^b^0.009
Mitochondrial damage (% abnormal MMP) (mean±SD)	25.9±16.4	24.6±16.4^a^	25.6±16.0^b^	29.0±17.1^a,b^	^a^0.006 ^b^0.04
Chromatin packaging (% CMA_3_ positive) (mean±SD)	56.1±15.2	57.7±15.0^a,b^	55.7±15.1^a,c^	52.9±15.6^b,c^	^a^0.01 ^b^<0.001 ^c^0.01
Apoptosis (%) (mean±SD)	19.2±15.2	19.1±8.0	19.3±7.9	19.3±7.8	0.85

### Cytochemical sperm parameters and age groups

**Figure 1:**
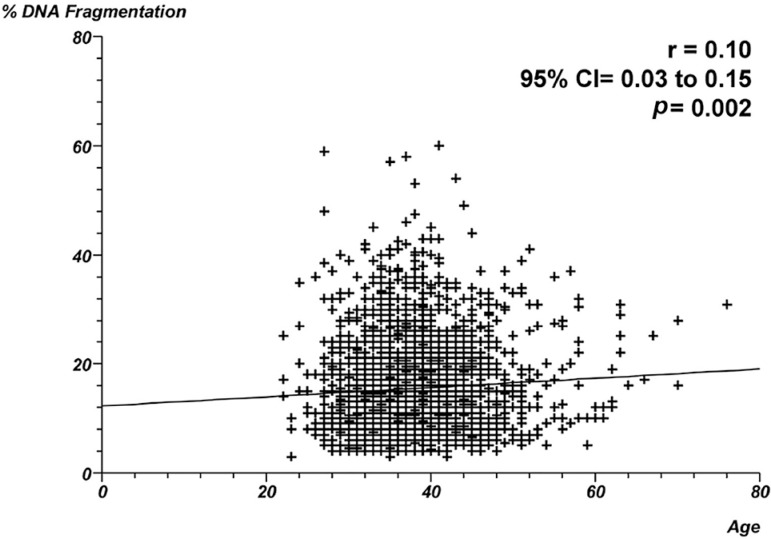
Correlation between male age and proportion of DNA fragmentation. Individual data points and the regression line are shown. Spearman's rank correlation coefficient = 0.10; *p*=0.002.

**Figure 2:**
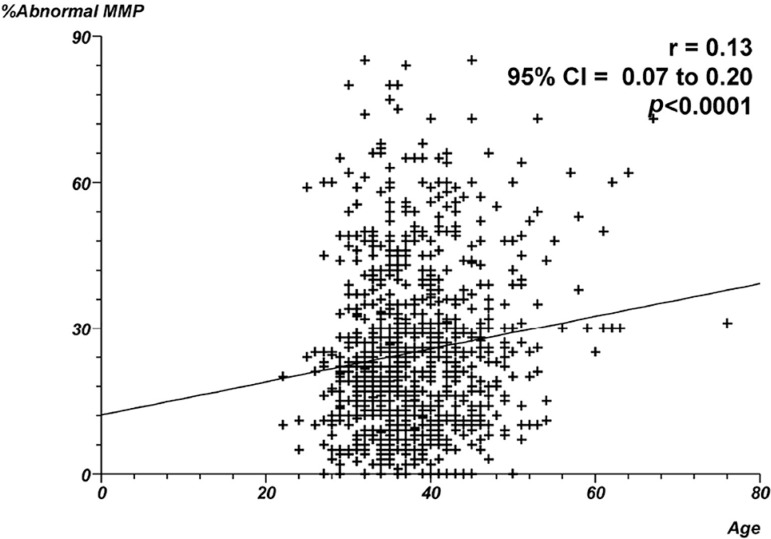
Correlation between male age and proportion of abnormal MMP. Individual data points and the regression line are shown. Spearman's rank correlation coefficient = 0.13; *p*<0.0001.

**Figure 3:**
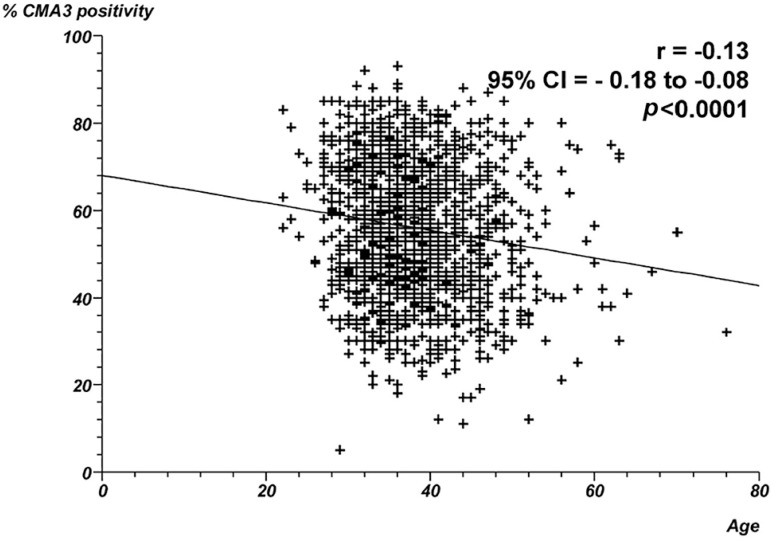
Correlation between male age and proportion of CMA positivity. Individual data points and the regression line are shown. Spearman's rank correlation coefficient =-0.13; *p*<0.0001.

**Figure 4:**
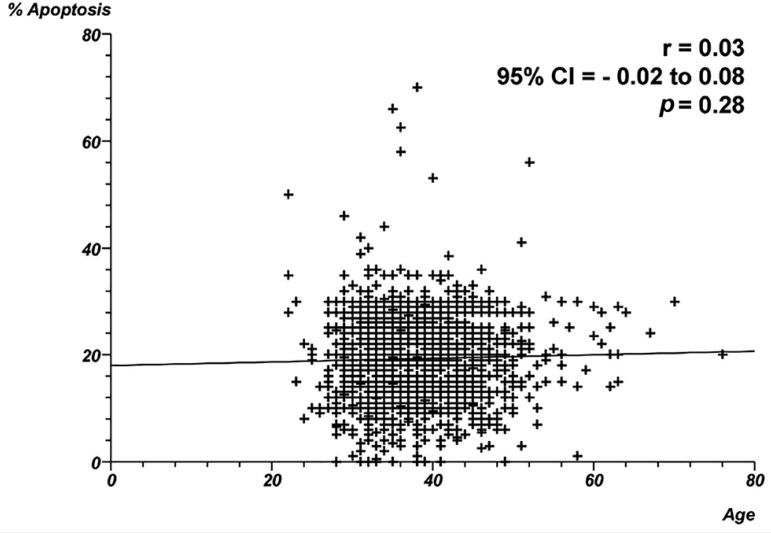
Correlation between male age and proportion of apoptosis. Individual data points and the regression line are shown. Spearman's rank correlation coefficient = 0.03; *p*=0.028.

Table 3 illustrates the cytochemical sperm parameters of 2178 patients divided into three groups based on male age. DNA damage increased with age. Patients aged ≤35 years presented statistically lower levels of DNA damage (14.7%) when compared to men aged 36-44 years (15.9%) and men aged ≥45 years (16.2%), *p*<0.05. Mitochondrial damage increased with age. Patients aged ≤35 years had a statistically lower proportion of abnormal MMP (24.6%) when compared to men aged 36-44 years (25.6%) and men aged ≥45 years (29.0%), *p*≤0.05. Abnormal chromatin packaging decreased as age increased. Patients aged ≤35 years had a statistically higher level of abnormal chromatin packaging DNA damage (57.7%) when compared to men aged 36-44 years (55.7%) and men aged ≥45 years (52.9%). There were no significant differences among the three groups in relation to age and apoptosis. Patients aged ≤35 years, 36−44 years, and ≥45 years had 19.1%, 19.3%, and 19.3% of spermatozoa with apoptosis, respectively. Figures 1 through 4 show the correlation between male age and cytochemical sperm parameters. The individual data points and the regression line demonstrated positive correlations between age and the proportion of DNA fragmentation - Spearman's rank correlation coefficient = 0.10; *p*=0.002 (Figure 1) - and age and the proportion of abnormal MMP (Figure 2) - Spearman's rank correlation coefficient = 0.13; *p*<0.0001. In contrast, a negative correlation was found between age and the proportion of CMA positivity (Figure 3) - Spearman's rank correlation coefficient = -0.13; *p*<0.0001. No correlation was found between age and proportion of apoptosis (Figure 4) - Spearman's rank correlation coefficient = 0.03; *p*=0.28.

## DISCUSSION

This study aimed to use a combination of assays to understand the effect of aging on spermatozoa DNA damage by testing physiologic processes, such as disulfide bond formation, chromatin protamination, and the events that measure functional endpoints, i.e., DNA denaturation and fragmentation. Our results showed that DNA fragmentation increased with age. These findings contrast against reports from other authors (Sun *et al.*, 1997; Winkle *et al.*, 2009; Colin *et al.*, 2010; Brahem *et al.*, 2011; Nijs *et al.*, 2011), in which no relationship was found between DNA fragmentation and age. Factors such as environmental exposure, sickness, differences in sample size, evaluation methods, and statistical analysis methods might have contributed to the differences between studies. However, our findings were consistent with the majority of studies that show a direct correlation between male age and DNA sperm damage, regardless of the technique used (TUNEL, Comet assay or high DNA stainability/HDS). However, most of these studies enrolled a small number (<300) of individuals (Wyrobek *et al*., 2006; Plastira *et al*., 2007; Morris *et al*., 2002; Singh *et al*., 2003; Schmid *et al*. 2007; Rybar *et al*., 2011; Das *et al*., 2013). With regard to larger population studies, Varshini *et al*. (2012) investigated 504 subjects and observed that patients over 40 years of age had significantly high levels of DNA damage. Moskovtsev *et al.* (2006) divided 1125 patients into five age groups and observed that DNA fragmentation significantly increased in individuals over 45 years of age when compared to all other age groups. Two additional studies confirmed the positive correlation between age and DNA fragmentation (Moskovtsev *et al*., 2007; 2009). Our earlier studies - one with 508 men and another with 1500 men - demonstrated clear increases in sperm DNA damage with age (Vagnini *et al*., 2007; Oliveira *et al*., 2014).

Although significant, the correlation between age and DNA fragmentation (*p*=0.002) might be considered weak (Spearman’s r=0.14), since it was similar to the values reported in the largest studies in the literature - r=0.29, *p*<0.001 (Moskovtsev *et al*., 2007); r=0.24, *p*<0.05 (Moskovtsev *et al.,* 2009). Others have reported stronger correlations between age and DNA fragmentation: Singh *et al.* (2003), r=0.56, *p*<0.001; Wyrobek *et al*. (2006) r=0.64-0.72, *p*<0.001; and Schmid *et al*. (2007), r=0.22, *p*<0.05 in alkaline conditions and r=0.06, *p*=0.58 in neutral conditions. Other authors have described it as a non-significant correlation (Sun *et al*., 1997). Unfortunately, not all studies have used this type of statistical analysis, which makes the interpretation of correlations a challenge.

Our data demonstrated a positive correlation between mitochondrial membrane potential (MMP) and age (*p*<0.001). Increased ROS production has been associated with altered mitochondrial membrane permeability with a likely subsequent loss of MMP, culminating with DNA fragmentation and cell death of both somatic cells and spermatozoa (Aitken *et al.*, 2012; Agarwal *et al*., 2014). The exact mechanisms related to advancing age remain to be unveiled, but the likely role of mitochondria in age-dependent decrease of male fertility might also be substantiated by sperm parameters. In fact, it has been recently demonstrated that sperm mitochondria do produce ROS, thus contributing to the onset of oxidative stress in the male gamete and possibly playing a key role in age-related reproductive pathophysiology (Koppers *et al*., 2008).

In fact, older men may produce more sperm with DNA fragmentation due to higher exposure to oxidative stress, defective sperm chromatin packaging, and disordered apoptosis (Agarwal & Said, 2003). The hypothesis is that the presence of DNA damage in mature spermatozoa is correlated with poor chromatin packaging (Ahmadi & Ng, 1999; Irvine *et al*., 2000). Double-stranded DNA breaks occur in the male germ during the process of chromatin packaging and are resolved during the spermatid stage of spermatogenesis (Sakkas *et al*., 1999). Abnormal chromatin packaging has been related to unresolved DNA breaks in immature human spermatozoa (Aitken *et al*., 2003; Aitken & De Iuliis, 2007). However, more evidence is required to support this idea.

Sperm packaging is of major importance. The tertiary structure of DNA carries epigenetic messages to the embryo, affects post-fertilization genome reprogramming, and impacts early embryonic development (Carrell, 2008; Rousseaux *et al*., 2008). However, DNA structure, i.e., condensation of DNA, is far less studied than age. Aniline or toluidine blue (Auger *et al*., 1990; Dadoune *et al.,* 1988; Hammadeh *et al*., 2001), which selectively stains lysine-rich histone proteins, and chromomycin A_3_ (CMA3), a guanine-cytosine-specific fluorochrome that competes with protamines for access to DNA (Sakkas *et al*., 1996), have been used to detect anomalies in protamine packaging. Unexpectedly, CMA_3_ staining revealed a significantly negative correlation between age and DNA packaging damage in our study. Although difficult to account for, one hypothesis regarding this phenomenon proposes that with age, there is an unbalanced redox system with higher caspase activation. This unbalance in free radicals might affect the disulfide bonds, making the fixation of protamines and chromomycin A_3_ difficult as they compete for the same receptors. Additionally, the pitfalls of the chromomycin A_3_ method cannot be ruled out. Belloc *et al*. (2009), using aniline blue, also demonstrated a negative correlation between age and chromatin packaging. DNA staining with this method showed a tendency to decrease chromatin damage with age (but the difference was not significant). In contrast, Nijs *et al*. (2011) showed a weak and significant positive correlation for chromatin packaging: a significantly higher proportion of spermatozoa had poor chromatin packaging and immature chromatin (measure by sperm chromatin structure assay/SCSA) in older individuals than the overall patient population (n=278) (*p*=0.035). This significant correlation, however, was lost when the patient population was split into three age groups (*p*>0.05).

The presence of apoptosis in ejaculated human sperm has received considerable attention, since defects in apoptosis have been proposed as an explanation for the generation of sperm DNA fragmentation (Agarwal *et al*., 2014; Sakkas & Alvarez, 2010). Ejaculated spermatozoa, particularly from infertile men, have been shown to display morphological and biochemical features that are typical of an apoptotic phenotype in somatic cells (Oehninger *et al*., 2003; Grunewald *et al.,* 2009). The deregulation of apoptosis is known to play a role in a number of disease processes, and it has been postulated that exacerbated or aberrant apoptosis might determine sperm dysfunction. Alternatively, in older men, the apoptotic functions of spermatogenesis might be defective, resulting in the production of more spermatozoa with fragmented DNA. However, in this study, we were unable to find correlations between age and apoptotic sperm cells. The proportion of spermatozoa detected by sperm phosphatidylserine (PS) translocation and the early apoptotic marker (annexin V) was similar between the three groups. In contrast, Collins *et al*. (2008) showed that advanced age per se was associated with a significantly increased expression of the early apoptotic biomarker in the ejaculate spermatozoa of healthy and proven fertile men.

In conclusion, sperm DNA damage seems to be influenced by the aging process. Although the influence of aging on sperm apoptosis was not observed, sperm DNA fragmentation increased with age and was apparently associated with mitochondrial damage. The age-related increase in sperm DNA damage suggests that postponing parenthood, not only in women but also in men, might jeopardize reproductive capacity. The increase in chromatin packaging with age is difficult to explain. The increase in chromatin packaging might represent a protective feature for DNA; however, a pitfall of the chromomycin A_3_ method cannot be ruled out. Additional studies must be performed to confirm the results concerning chromatin packaging (chromatin protamination) in sperm.
